# Consumption of hot beverages and foods and the risk of esophageal cancer: a meta-analysis of observational studies

**DOI:** 10.1186/s12885-015-1185-1

**Published:** 2015-06-02

**Authors:** Yawen Chen, Yeqing Tong, Chen Yang, Yong Gan, Huilian Sun, Huashan Bi, Shiyi Cao, Xiaoxv Yin, Zuxun Lu

**Affiliations:** School of Public Health, Tongji Medical College, Huazhong University of Science and Technology, Wuhan, Hubei China; Center for Disease Control and Prevention of Hubei Province, Wuhan, Hubei China

**Keywords:** Hot, Beverage, Food, Esophageal cancer, Meta-analysis

## Abstract

**Background:**

Previous studies have mostly focused on the effects of specific constituents of beverages and foods on the risk of esophageal cancer (EC). An increasing number of studies are now emerging examining the health consequences of the high temperature of beverages and foods. We conducted a meta-analysis to summarize the evidence and clarify the association between hot beverages and foods consumption and EC risk.

**Methods:**

We searched the PubMed, Embase, and Web of Science databases for relevant studies, published before May 1, 2014, with the aim to estimate the association between hot beverage and food consumption and EC risk. A random-effect model was used to pool the results from the included studies. Publication bias was assessed by using the Begg test, the Egger test, and funnel plot.

**Results:**

Thirty-nine studies satisfied the inclusion criteria, giving a total of 42,475 non-overlapping participants and 13,811 EC cases. Hot beverage and food consumption was significantly associated with EC risk, with an odds ratio (OR) of 1.82 (95% confidence interval [CI], 1.53–2.17). The risk was higher for esophageal squamous cell carcinoma, with a pooled OR of 1.60 (95% CI, 1.29–2.00), and was insignificant for esophageal adenocarcinoma (OR: 0.79; 95% CI: 0.53–1.16). Subgroup analyses suggests that the association between hot beverage and food consumption and EC risk were significant in Asian population (OR: 2.06; 95% CI: 1.62-2.61) and South American population (OR: 1.52; 95% CI: 1.25-1.85), but not significant in European population (OR: 0.95; 95% CI: 0.68-1.34).

**Conclusions:**

Hot beverage and food consumption is associated with a significantly increased risk of EC, especially in Asian and South American populations, indicating the importance in changing people’s dietary habits to prevent EC.

## Background

Esophageal cancer (EC) is the eighth most common cancer in the world and ranks six among all cancers in mortality [1]. Many studies have shown that dietary habits are significantly correlated with the occurrence of EC [2,3], most of which linking specific constituents of beverages and foods to EC. For example, Polyphenols in green tea was found to inhibit esophageal tumorigenesis [4], whereas maté infusion and caffeine appeared to induce mutagenic effects [5]. An increasing number of studies have investigated the possible relationship between the temperature of beverages and foods and EC risk [6-8], since recurrent thermal injuries to the esophageal mucosa owing to the consumption of hot drinks or foods has long been considered a risk factor for EC [9].

Hot beverage consumption could substantially increase the intraesophageal temperature, depending on the initial drinking temperature. An animal study showed that the structure and the function of the esophageal epithelium were damaged by heat stress even [10]. However, epidemiological evidence on the causal relationship between the temperature of beverages and foods and EC is not well established. Research on the relationship was often done as a component of larger studies that focused on specific beverage or food gradients, and the results varied greatly across studies. Some studies found no association between hot beverages and foods and EC risk [11-13], arguing that the oral cavity could modulate the heat, and the temperature could fall too rapidly to cause injury to the esophageal mucosa [14]. But many other studies reported that the intake of hot beverages and foods increased EC risk [11,15,16].

In 2009, Islami and colleagues [9] reviewed fifty nine studies and found that over half of the studies showed statistically significant increased risk of EC associated with higher temperature of beverage and food intake. However, the authors did not use quantitative techniques to compute summary estimates of the risk, and the review is outdated. Therefore, we conducted this meta-analysis to ascertain the association between hot beverage and food consumption and EC risk more precisely, relying on all available evidence up-to-date, and to identify the potential factors affecting this association.

## Methods

### Search strategy

This meta-analysis was conducted according to the checklist of the Meta-Analysis of Observational Studies in Epidemiology Guideline [17]. We searched PubMed, Embase, and Web of Science databases from inception to May 1, 2014 for all epidemiological studies on hot beverage and food consumption in relation to EC risk, using the string ‘(esophageal OR oesophageal) AND (cancer OR carcinoma OR neoplasm) AND (tea OR maté OR coffee OR beverage OR liquid OR alcohol OR food OR diet)’. In addition, we scrutinized the reference lists from retrieved articles to identify other relevant studies.

### Inclusion criteria

Studies were considered eligible for inclusion if they met the following criteria: (1) the study was a case–control or cohort study design, (2) it was published in English, (3) the exposure was hot beverage or food consumption, (4) the outcome of interest was EC, and (5) the study reported the odds ratio (OR) or relative risk (RR) with 95% confidence intervals (CIs) for the association between hot beverages or foods and EC risk or provided sufficient data to calculate them.

### Date extraction

We extracted the following data from each retrieved article: name of first author, publication year and country of study, study design, specific outcomes, characteristics of study population, number of cases and participants, exposure type, exposure measurement, outcome assessment, comparison categories, OR or RR and corresponding 95% CI, and confounding factors adjusted in the analyses. Data from included studies were independently extracted by two authors (Y.W.C and Y.C), and disagreements were resolved through discussion with the third reviewer (Z.X.L).

### Quality assessment

Two independent reviewers (Y.W.C and C.Y) evaluated the quality of the included studies by the Newcastle-Ottawa Scale [18], which was a nine-point scale that allocated points based on the selection process (0-4points), the comparability (0–2 points), and the assessment of outcomes of study participants (0-3points). We assigned scores of 0–3, 4–6, and 7–9 for low, moderate, and high quality of studies, respectively.

### Statistical analysis

Random-effects model was used to estimate the summary ORs or RRs for the association between hot beverage and food consumption and EC risk. Taking the subjectivity of differentiating between hot and very hot into account, we used the specific OR for standardized category (hot and very hot) versus reference category (cold and warm) of beverage and food consumption. We defined exposure as hot beverages and foods (standardized category, preference for high-temperature foods and drinks, often consuming of them) versus non-hot beverages and foods (all other combinations). If studies had partly overlapped subjects, only the one with a larger sample size was selected for the analysis. If a study reported results for different beverages and foods separately, those beverage/food specific results were regarded as separate reports on the relationship between temperature and EC risk. One study [11] contained 4 kinds of drinks, and was, therefore, accounted as four independent reports. Another study [19] reporting tea, water and food was regarded as three reports. Two studies [13,20] conducted in two different areas of China were considered as two reports respectively, and another study [16] including two large multicenter case–control studies was treated as two reports.

Statistical heterogeneity among studies was evaluated using the *I*^*2*^ statistic, where values of 25%, 50% and 75% represent cut-off points for low, moderate and high degrees of heterogeneity, respectively [21]. To assess the heterogeneity across all included studies, the study location (Asia, South America, Europe, Africa), study setting (population-based, hospital based), study quality (≥7,<7),type of EC (esophageal squamous cell carcinoma (ESCC), esophageal adenocarcinoma (EAC)), and sample size (≥1000,<1000) were further examined using meta-regression. In sensitivity analyses, we conducted leave-one-out analyses [22] for each study to examine the magnitude of influence of each study on pooled risk estimates. Subgroup analyses by age, sex, study location, hot beverage and food categories, study quality, smoking and alcohol intake, study setting, outcome assessment and exposure assessment were conducted to examine the robustness of the primary results. Publication bias was assessed using the Begg test [23], the Egger test [24] and funnel plot. All statistical analyses were performed using STATA version 11.0 (Stata Corp, College Station, Texas, USA). All tests were two sided with a significance level of 0.05.

## Results

### Literature search

**Figure 1:**
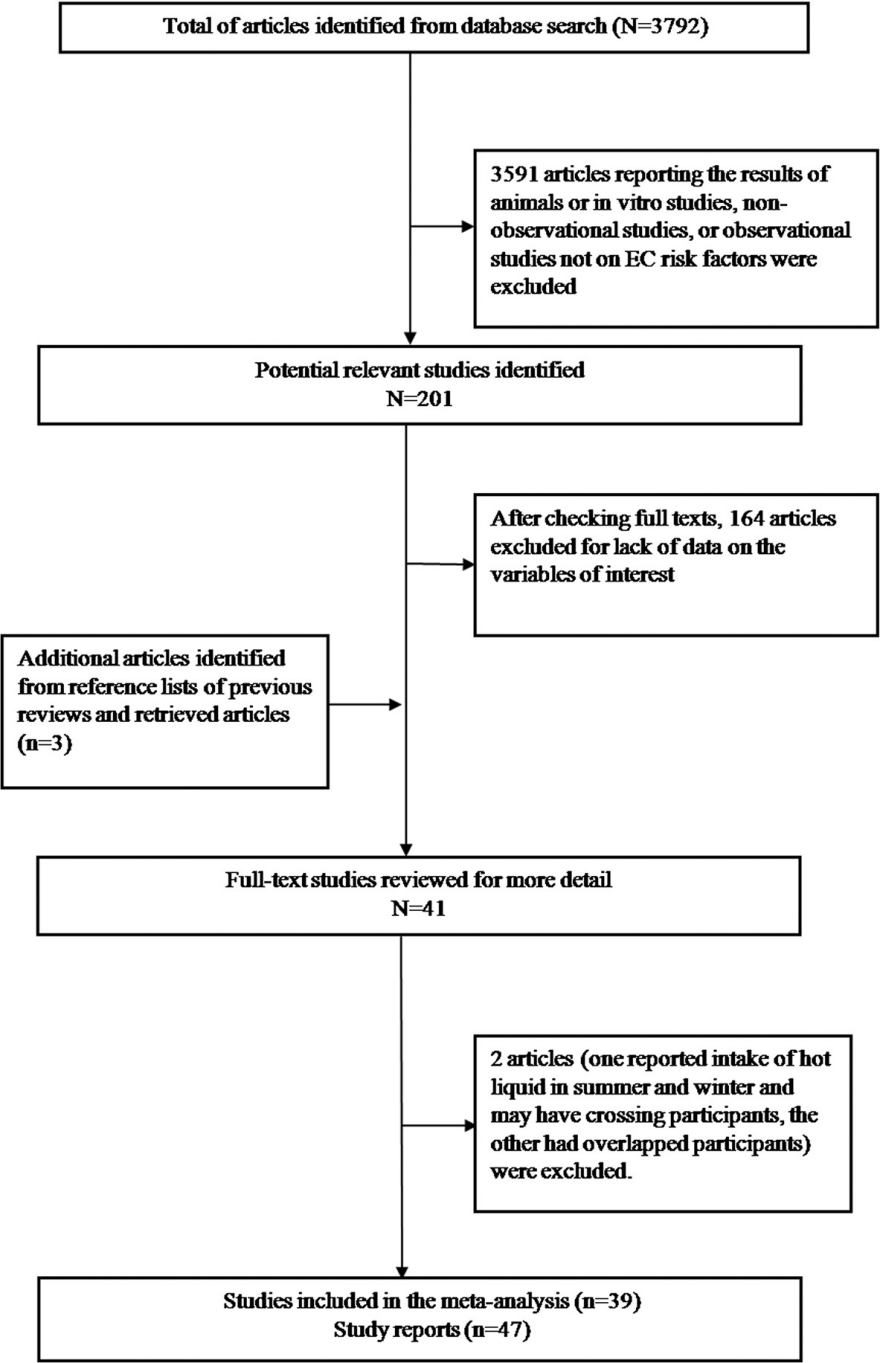
**Study selection process.**

The search identified a total of 3780 unique articles from PubMed, Google scholar, and Web of Science databases, of which 189 articles were identified as potentially relevant. After retrieving and reviewing the full text, we determined that 39 studies met our inclusion criteria. The process of study selection is shown in Figure 1.

### Study characteristics

Table 1 shows the main characteristic of the 39 included studies. These studies were published between 1979 and 2014, all of which with case–control design. The sample sizes of studies ranged from 143 to 4,118 with a total of 42,475 subjects. The number of EC cases diagnosed in the studies ranged from 47 to 1,310, with a total of 13,811 reported EC cases. Seventeen studies were conducted in China [13,19,20,25-38], six in Uruguay [11,16,39-42], three in Argentina [11,16,43], three in Brazil [11,16,44], three in Paraguay [11,16,45], three in India [46-48], three in Iran [15,49,50], two in British [8,51], one in Australia [12], one in Sweden [7], one in Greece [6], one in Kenya [52], and one in Japan [53]. Thirty studies reported results for men and women together, four reported the results for men and women separately, and three reported results for men only and two for women only. Three studies reported results separately by type of EC. Two studies were deemed high quality, 36 moderate quality studies, and one low quality study. The average quality score for all included studies was 5.00.

**Table 1:** **Characteristics of studies included in the meta-analysis**

**Study source**	**Study design**	**Sex**	**Age at baseline(years)**	**No of cases**	**No of participants**	**Exposure assessment**	**Outcome assessment**	**Exposure categories used in meta-analysis**	**Adjustment for confounders**	**Quality assessment**
Islami et al., [15], northern Iran	Population based case–control	F/M	Cases:64.5 ± 10.1 controls:64.3 ± 10.4	300	871	Interviews	Endoscopy and biopsy samples	Tea: hot/very hot vs. warm	Ethnicity, daily vegetable intake, alcohol consumption, tobacco or opium use, duration of residence in rural areas, education level, and car ownership	7
Lin et al., [35],Southern China	Hospital based case–control	F/M	Cases:54.5 ± 4.9 controls:52.5 ± 3.7	213	426	FFQ	Endoscopically and histologically confirmed	Beverage: hot/very hot vs. lukewarm	Age, sex, educational status, smoking, drinking, body mass index, vegetable and fruit	6
Rolon et al., [45], Paraguay	Hospital based case–control	F/M	≤45:33 46–55:89 56–65:188 ≥ 66:202	131	512	Interviews	Cytology, histology, or radiology	Maté: very hot vs. warm/hot	design variables, lifetime cigarette consumption, and lifetime alcohol consumption	6
Stefani et al., [41], Uruguay	Hospital based case–control	F/M	40-89	166	830	Questionnaire	Histologically verified	Maté: hot/very hot vs. warm	NR	6
Castelletto et al., [43], Argentina	Hospital based case–control	F/M	≤54:80 55–64:129 65–74:127 ≥ 75:57	131	393	Questionnaire	Histological diagnosis	Maté: hot/very hot vs. warm	Education, average number of cigarettes/day, alcohol consumption (ml/day), the design variables	6
Castellsagu´e et al., [11], south America	Hospital based case–control	F/M	64.0(mean)	830	2609	Interviews with structured questionnaire	Histologically confirmed or a cytological or radiological diagnosis	Maté, tea, coffee, coffee with milk: hot/very hot vs. cold/warm	Age group, hospital, residency, years of education, average number of cigarettes/day, average amount of pure ethanol/day and gender	6
Ibiebele et al., [12], Australia	Population based case–control	F/M	18-79	521	1965	FFQ	Registries	Tea/coffee: hot/very hot vs. lowest	Age, gender; cumulative history of smoking in pack years, lifetime mean alcohol intake; heartburn and acid reflux symptoms, body mass index, educational status, aspirin use in previous 5 years, total fruit and vegetable intake and total energy intake in kilojoules	6
Szyman´ska et al., [44], Latin America	Hospital based case–control	F/M	NR	71	228	Lifestyle questionnaire	ICD-O classification	Maté: hot/very hot vs. cold/warm	NR	5
Chen et al., [26], Southern China	Hospital based case–control	F/M	Cases:54.6 ± 6 controls:54.0 ± 7	87	267	Self-designed structured questionnaire	Histologically confirmed	Tea: hot/very hot vs. warm	NR	5
Sewram et al., [42], Uruguay	Hospital based case–control	F/M	35-85	295	685	Questionnaire	Registries	Maté: very hot vs. warm/hot	Amount consumed, and duration of mate´ consumption	5
Tang et al., [19], China	Hospital based case–control	F/M	61 ± 11.4	359	739	Structured questionnaire	Medical records and pathology reports	Tea, water, food: high vs. low or mild	Age, gender, education level, body mass index, smoking status, alcohol drinking, family history of cancer in first-degree relatives, daily intake of vegetables and daily intake of fruit	6
Stefani et al., [40], Uruguay	Hospital based case–control	F/M	40-89	234	702	Questionnaire	Microscopically confirmed	Maté: hot/very hot vs. warm	NR	5
Wu et al., [20], China	Population based case–control	F/M	NR	665	2000	Pretested standardized epidemiologic questionnaire	registry	Tea: high vs. normal	NR	5
Sharp et al., [8], England and Scotland	Population based case–control	F	<75(<80in Trent)	156	312	Interviews	Histologically confirmed	Tea/coffee: hot/burning hot vs. warm	NR	5
Terry et al., [7], Sweden	Population based case–control	F/M	<80	189	1004	interviews	Histologically confirmed	Tea/coffee: hot/very hot vs. cold/lukewarm	Age, gender, body mass index, cigarette smoking, socioeconomic status presence of Gastro-oesophageal reflux symptoms, frequency quartiles of hot beverage consumption, and quartiles of alcohol, fruit and vegetables, and energy consumption	5
Lubin et al., 2014, South America, [16]	Case–control	F/M	35-85	1310	4118	Questionnaire	Medical records	Maté: hot/very hot vs. warm vs.	NR	5
Wang et al. [37], China	Population based case–control	F/M	Mean: cases 61.51 controls 60.75	355	763	Structured questionnaire	Pathologically diagnosed	Food: hot vs. warm	Age (continuous), marital status and education years	7
Phukan et al., [47], India	Hospital based case–control	F/M	Case:55.0 ± 8.1 control:54.5 ± 7.8	502	1511	Investigation	Histopathologically confirmed	Food: hot vs. moderate	Education, income, chewing betel nut and tobacco, smoking, and alcohol use	4
Wu et al., [13], China	Population based case–control	F/M	<50: 67 50–59:219 60–69: 428 70–79:295 ≥ 80:53	531	1062	Pre-tested standardized questionnaires	Cancer registration database	Food: hot vs. normal	NR	4
Gao et al., [29], China	Population based case–control	F/M	30-74	902	2454	Structured questionnaire	Registry	Hot soup or porridge: hot/burning hot vs. cold/neither cold nor hot	Age, education, birthplace, tea drinking, cigarette smoking, alcohol drinking and consumption of preserved foods, vegetables and fruit	6
Hu et al., [32], China	Hospital based case–control	F/M	35-69	196	588	Interviews	Histopathologically confirmed	Gruel: hot/scalding vs. lowest	Smoking, alcohol, income and occupation	4
Garidou et al., [6], Greece	Hospital based case–control	F/M	<60: 79 60–69:103 ≥ 70: 117	99	299	Questionnaire	Histologically confirmed	Preferrable temperature: very hot vs. cold to hot	Gender, age, birthplace, schooling, height, analgesics, coffee drinking, alcohol intake, tobacco smoking and energy intake	4
Cheng et al., [51], British	Population based case–control	F	Cases:65.9 controls:65.3	74	148	Questionnaire and interview	Histologically confirmed	Preference tea or coffee: hot very/burning hot/hot vs. warm	NR	4
Hanaoka et al., [53], Japan	Hospital based case–control	M	Under 85 years old	141	282	Structured questionnaire	Confirmed histologically by biopsy examination	Preference for high = temperature food and drink: like vs. dislike	Alcohol consumption (g/week)	4
Srivastava et al., [48], India	Case–control	F/M	NR	170	340	Pretested. Semi-structured questionnaires	Endoscopic, radiological and histopathological assessments	Food: hot vs. warm	NR	4
Stefani et al., [39], Uruguay	Hospital based case–control	F/M	40-49:45 50–59:120 60–69:207 70–79:183 80–89:45	200	600	Questionnaire	Newly diagnosed and microspically confirmed	Maté temperature: hot/very hot vs. warm	NR	5
Cheng et al., [27], Hong Kong of China	Case–control	F/M	<45:40 45–54:246 55–64: 722 65–74:696 > =75: 294	400	1998	Interviews with structured questionnaire	Histologically confirmed diagnoses	Preference for hot drinks or soups: yes vs. no	Adjusted for age and education, place of birth, green leafy vegetables, pickled vegetables, citrus fruits, tobacco and alcohol	4
Gao et al., [30], China	Population based case–control	F/M	30-74	653	1965	Structured, standardized questionnaire	Registry	Burning-hot fluids:yes vs. no	NR	5
Cook-mozaffari et al., [49], Iran	Case–control	F/M	NR	344	1032	Questionnaire	Registry	Drinking of hot tea: yes vs. no	NR	4
Guo et al.,[31], China	Nested case–control	F/M	40-69	640	3840	Structured questionnaires	X-ray films and cytological, pathological, surgical specimens	Hot liquid:≥1 vs.0	Years of smoking and cancer history in first degree relatives	6
Ke et al., [34], China	Hospital based case–control	F/M	29-82	1064	2168	Questionnaires and FFQ	Histologically confirmed	Hot Congou drinkers vs. non-hot Congou drinkers	NR	5
Patel et al., [52], Kenya	Hospital based case–control	F/M	Mean:56.1	159	318	Questionnaires	NR	Take hot beverages: yes/no	NR	4
Hung et al., [33], Taiwan of China	Case–control	M	Mean:62.4	267	697	Interviews according to standardized questionnaire	Histologically confirmed	Hot drink or soup: 3+ time per day vs. <3 time per day	Adjusted for age, educational levels, ethnicity, source of hospital, smoking, alcohol drinking and areca nut chewing	4
Chen et al., [25], Taiwan of China	Hospital based case–control	M	40-50:284 51–60::291 61–70 :314 > 70:209	274	922	Interviews	Newly histologically diagnosed	Hot drink or soup: > = 1 time/d vs. <1time/d	Adjusted for age, educational levels, ethnicity, source of hospital, smoking, alcohol drinking, and areca nut chewing	4
Gao et al., [28],China	Case–control	F/M	51-65	600	2114	Questionnaires	Histologically confirmed	Scalding hot food: daily vs. weekly/never/monthly/seldom	NR	4
Sun et al., [36],China	Population based case–control	F/M	Cases:61.21 ± 8.95 Controls:60.84 ± 8.90	250	1000	Questionnaires	Cancer registration database	Hot foods: often vs. sometimes	NR	6
Yang et al., [38], China	Case–control	F/M	Cases:58.1 (8.5) Controls:57.9 (8.8)	185	370	Questionnaires	Histologically diagnosed within half a year	Hot foods: often vs. Rarely/occasionally	NR	6
Jessri et al., [50], Iran	Hospital based case–control	F/M	40-75	47	143	Structured pre-tested questionnaires	Histologically-confirmed	Food and beverages temperature: hot vs. warm/cold	NR	4
Khan et al., [46], India	Case–control	F/M	Case:54.3(7.6) Control:58.1(8.3)	100	200	Questionnaires	Histologically-confirmed	Degree of hotness: hot vs. warm	NR	3

Age presents the range with Mean (SD). Abbreviations: NR = not reported; F = female; M = male.

### Hot beverage and food consumption and the risk of esophageal cancer

**Figure 2:**
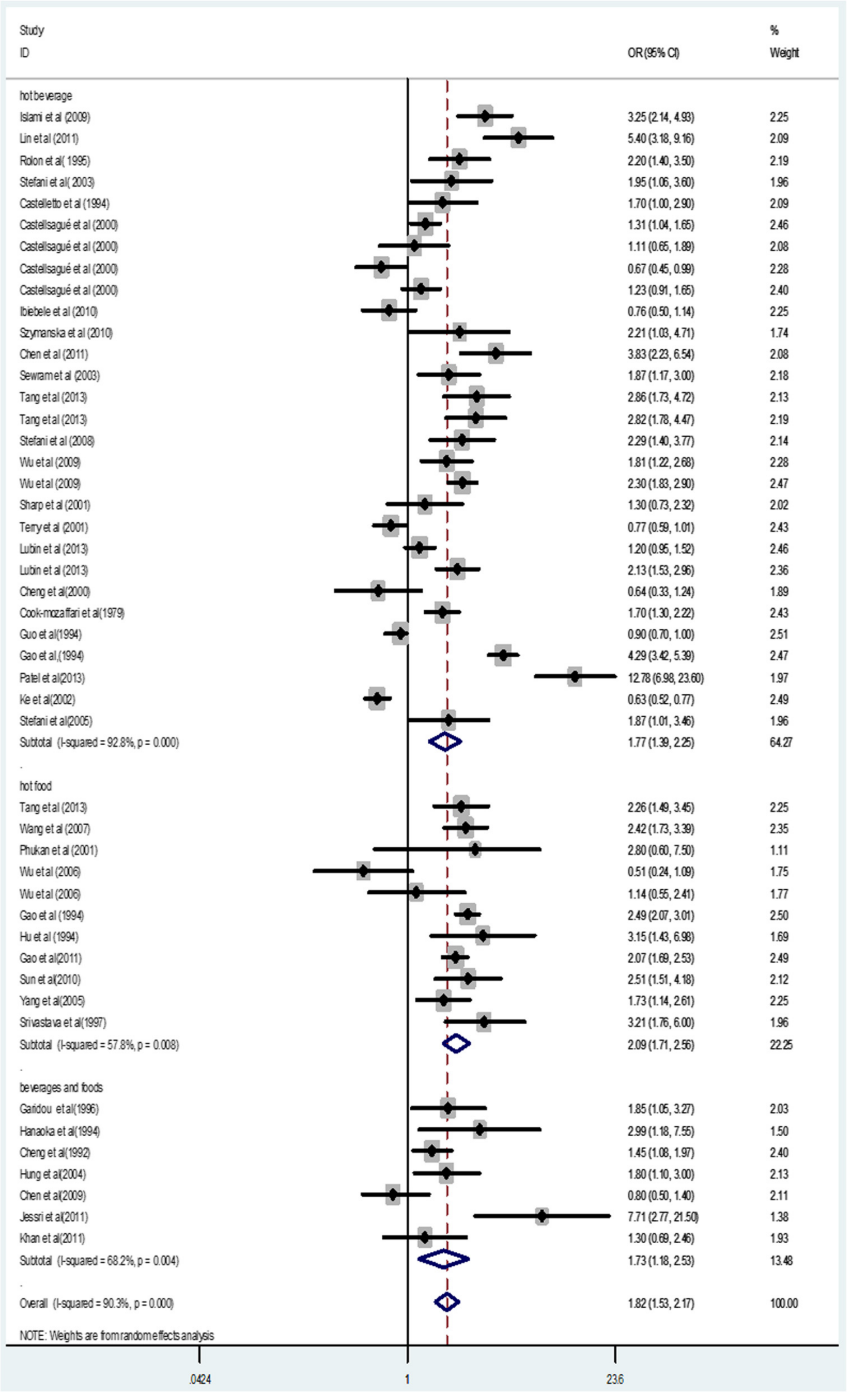
**Forest plot of odds ratios from 39 studies linking hot beverage and food consumption and the risk of esophageal cancer.**

The results from the random-effects meta-analysis of hot beverage and food consumption and the risk of EC were shown in Figure 2. Thirty-two of 47 independent reports from 39 studies suggested a positive relation between hot beverage and food consumption and EC risk. The pooled OR was 1.77(95% CI, 1.39–2.25), with a high heterogeneity (*I*^*2*^ = 92.8%, *p* = 0.001); the pooled OR was 2.09(95% CI, 1.71–2.56, *I*^*2*^ = 57.8%, *p* = 0.008); and the pooled OR of EC risk in relation to hot beverage and food consumption was 1.73(95% CI, 1.18–2.53, *I*^*2*^ = 68.2%, *p* = 0.004).

### Subgroup analysis

Table 2 showed the results based on subgroup analyses, which were to examine the stability of the primary results and explore the resources of potential heterogeneity. The associations between hot beverage and food consumption and the risk of EC were similarly significant in subgroup analyses, with the exception of EAC (OR = 0.79, 95% CI = 0.53–1.16, *I*^*2*^ = 50.30%, *P* = 0.110) and European population (OR = 0.95, 95% CI = 0.68–1.34, *I*^*2*^ = 62.40%, *P* = 0.031).

**Table 2:** **Subgroup analysis of odds ratio of hot beverages and foods and esophageal cancer**

	**No of reports**	**OR**	**(95% CI)**	**I** ^**2**^	**P for heterogeneity**
Sex					
Men	8	2.36	1.53–3.65	87.60%	0.001
Women	7	2.45	1.51–3.98	85.60%	0.001
Combined	37	1.78	1.49–2.16	89.30%	0.001
Type of EC					
ESCC	26	1.60	1.29–2.00	88.70%	0.001
EAC	4	0.79	0.53–1.16	50.30%	0.110
NR	20	2.35	1.90–2.91	80.70%	0.001
Study quality					
Score ≥ 7	2	2.73	2.06–3.62	12.90%	0.284
Score < 7	45	1.78	1.49–2.14	90.40%	0.001
Study location					
Asia	28	2.06	1.62–2.61	91.70%	0.001
South America	13	1.52	1.25–1.85	66.70%	0.001
Europe	5	0.95	0.68–1.34	62.40%	0.031
Africa	1	12.78	6.95–23.5	.	0.001
Measurement domain					
Temperature categories	33	1.84	1.54–2.21	83.80%	0.001
Whether consuming or not	5	2.14	0.94–4.88	98.30%	0.001
Preference	4	1.44	0.88–2.35	66.30%	0.031
Frequency	5	1.71	1.24–2.36	68.90%	0.012
Beverages and foods domain					
Tea	8	1.88	1.16–3.07	94.30%	0.001
Mate	10	1.72	1.43–2.07	47.50%	0.046
Foods	11	2.09	1.71–2.56	57.80%	0.008
Others	18	1.73	1.19–2.49	93.70%	0.001
Controlling age in models					
Yes	17	1.6	1.24–2.07	88.50%	0.001
No	30	1.98	1.55–2.52	91.30%	0.001
Controlling smoking in models					
Yes	29	1.61	1.26–2.07	89.30%	0.001
No	27	2	1.56–2.55	90.50%	0.001
Controlling alcohol intake in models					
Yes	19	1.56	1.21–2.02	88.00%	0.001
No	28	2.03	1.59–2.59	91.60%	0.001
Study setting					
Population	14	1.52	1.07–2.16	94.1%	0.001
Hospital	24	2.10	1.56–2.82	89.8%	0.001
NR	9	1.73	1.44–2.06	59.7%	0.040
Exposure assessment					
Interview	14	1.33	1.03–1.71	80.0%	0.001
Questionnaire	33	2.07	1.67–2.57	91.5%	0.001
Outcome assessment					
Histology	30	1.68	1.36–2.07	88.2%	0.001
Record	17	1.90	1.50–2.41	86.3%	0.001

Abbreviations: EC = esophageal cancer; ESCC = esophageal squamous cell carcinoma; EAC = esophageal adenocarcinoma; NR = not reported.

### Sensitivity analysis and meta-regression

We excluded each study in turn and pooled the results of the remaining included studies. The positive association was not materially changed upon the exclusions, with a pooled OR range from 1.75 (95% CI, 1.47 to 2.07; *P =* 0.001) to 1.87(95% CI, 1.58 to 2.20; *P* = 0.001), which indicates that the overall result was not significantly influenced by any individual studies.

Our meta-regression analysis reveals that the study location (*P* = 0.001), the type of EC (*P* = 0.047) and sample size (*P* = 0.033) were significant sources of heterogeneity. Study location alone explained 34.39% of the τ^2^ in the meta-regression; type of EC explained 12.97%; and sample size explained 8.99%. The results were shown in Table 3.

**Table 3:** **Meta-regression analysis**

**Variable**	**Coefficient**	**Standard error**	***P*** **value**	**95% CI**
Study location				
Asia	−1.833	0.568	0.002	−2.979–-0.688
South America	−2.109	0.578	0.001	−3.273–-0.945
Europe	−2.582	0.607	0.001	−3.807–-1.357
Type of EC	−0.678	0.327	0.047	−1.348–-0.009
Sample size	−0.403	0.183	0.033	−0.771–-0.034

Abbreviations: EC = esophageal cancer.

### Publication bias

**Figure 3:**
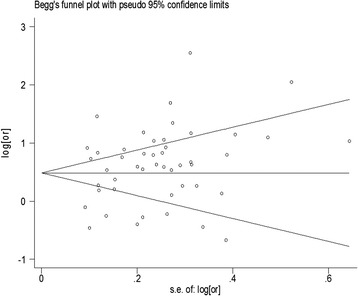
**Funnel plot of hot beverages and foods and the risk of esophageal cancer.**

Visual inspection of funnel plot did not identify substantial asymmetry (see Figure 3). The Begg rank correlation test and the Egger linear regression test indicated no evidence of publication bias across included studies (Begg test *Z* = 0.59, *P* = 0.557; Egger test *t* = 1.58, *P* = 0.121).

## Discussion

In this large pooled analysis of 42475 participants (13811 EC cases) from 39 case–control studies, we confirmed a positive association between hot beverage and food consumption and EC risk. Individuals who usually have beverages and food served very hot or hot were almost twice likely to develop EC than individuals who usually have beverages and foods served warm or cold. Our subgroup analyses show that the results held true across various populations despite significant heterogeneity.

Our meta-analysis shows that the consumption of hot beverages and foods are significantly associated with ESCC (OR, 1.60; 95% CI, 1.29–2.00) but not with EAC (0.79, 95% CI, 0.53–1.16). A large body of observational evidence suggests that the risk factors for ESCC and EAC may be different. For example, alcohol intake is a strong and well established risk factor for ESCC but it is not associated with EAC [54]; a high body mass index (BMI) is associated with an increased risk of EAC but a decreased risk of ESCC [55]; ESCC is strongly associated with high-level exposure to tobacco smoking in Western populations [54,56], whereas EAC is associated with gastro-esophageal reflux disease and Barrett’s esophagus [57]. More studies are needed to explore why hot beverage and food consumption is associated with an increased risk for ESCC but not EAC.

Another notable finding is that hot beverage and food consumption appears not to be a risk factor for EC in European population (OR, 0.95; 95% CI, 0.68–1.34). The result might be ascribed to the small sample size (3,728 participants and 1,039 EC cases) or the unique dietary habits of Europeans. A previous study noted that Europeans tend to add cold milk to the exposure beverages, tea or coffee before consumption [12], which may cause people say they drink hot actually only warm and result in substantial difference between the temperature perceived by drinkers and the actual temperature of their drinks.

It is conceivable that hot beverages and foods may cause thermal injury to the esophageal mucosa, and there are several biological mechanisms through which thermal injury in general could increase the risk of EC. Inflammatory processes associated with chronic irritation of the esophageal mucosa caused by local hyperthermia could stimulate the endogenous formation of reactive nitrogen species and nitrosamines [58]. This hypothesis is supported by a high rate of somatic G to A transitions in CpG dinucleotides of the *TP53* gene in esophageal tumor samples from geographical areas in which drinking hot beverages is considered an important risk factor for EC [59-62]; these mutations may indicate increased nitric oxide synthase activity in tumors [63]. The barrier function of the esophageal epithelium can be impaired by thermal injury, which may increase the risk of damage from exposure to intraluminal carcinogens [10], such as polycyclic aromatic hydrocarbons. Elevated temperatures could also accelerate metabolic reaction, including those with carcinogenic substances in tobacco and alcohol [64]. In fact, the association between consuming hot drinks and the occurrence of precancerous lesions of the esophagus has been repeatedly reported [65-67]. In addition, dietary deficiencies may weaken the esophageal tissue because of the constant irritation, which may act as a predisposing factor for EC [47]. It has also been postulated that contact of hot liquid and food with the esophageal mucosa could increase gastric reflux, causing further damage from gastric acid [68]. One review proposed that the overproduction of prostaglandin E_2_ and leukotriene B_4_ as well as overexpression of their receptors are major factors in exacerbating inflammation and oxidative stress, which is the main pathogenesis associated with EAC [57]. The result from our meta-analysis of epidemiological studies is consistent with these biomedical research findings and postulations.

All the original studies used in our meta-analysis are of case–control study design, which is particularly vulnerable to potential biases (both selection bias and information bias). The included studies were conducted among different populations, mostly along with various categorizations of beverages and foods, which could confound our analysis on the specific link between the temperature of beverages and foods and the risk of EC. Lastly, the study relied on self-reported consumption of hot beverages and foods; as a result, the categorization of “hot or very hot” versus “cold or warm” is subject to reporting bias. In addition, the limited information provided in the included studies ruled out the possibility of conducting a dose–response analysis. Nonetheless, this is the first meta-analysis to systematically quantify the association between hot beverage and food consumption and EC risk, and the results of our study are of broad interest to medical science and the public since consumption of beverages such as tea, coffee, and maté are prevalent worldwide [64,69,70] and many people prefer to drink them at a high or very high temperature [15,71].

In the light of our findings, certain factors should be considered in future studies. Large prospective studies are needed to investigate the association of hot beverage and food consumption with both EC risk and the type of EC, not only because of the different ESCC and EAC risk factors but also the rapid changes in incidence of EAC [12]. In addition, measuring the actual temperature of hot beverage and food would provide dose–response data that would allow for evaluation of the relationship with EC risk more precisely. Finally, confounding factors, such as BMI, smoking, alcohol intake, and socioeconomic status, should be adjusted to allow dissection of the actual influence of hot beverage and food on EC, thereby providing provide stronger research-based evidence.

## Conclusions

In summary, our meta-analysis shows that hot beverage and food consumption is associated with a significantly increase in the risk of EC, especially in Asian and South American populations. Given that hot beverages and foods are prevalent in modern society, the results of our meta-analysis have important implications for cancer etiology research as well as applications in health education and clinical practice.

### Ethics approval

Ethical approval is not required for this review.
